# Investigating variations of precipitation concentration in the transitional zone between Qinling Mountains and Loess Plateau in China: Implications for regional impacts of AO and WPSH

**DOI:** 10.1371/journal.pone.0238709

**Published:** 2020-11-05

**Authors:** Ci Li, Hongbo Zhang, Vijay P. Singh, Jingjing Fan, Xiaowei Wei, Jiantao Yang, Xingchen Wei

**Affiliations:** 1 School of Water and Environment, Chang’an University, Xi’an, China; 2 Key Laboratory of Subsurface Hydrology and Ecological Effect in Arid Region, Ministry of Education, Chang’an University, Xi’an, China; 3 Department of Biological and Agricultural Engineering, Texas A&M University, College Station, Texas, United States of America; 4 Zachry Department of Civil and Environmental Engineering, Texas A&M University, College Station, Texas, United States of America; 5 National Water Center, UAE University, Al Ain, UAE; 6 School of Water Conservancy and Hydroelectric Power, Hebei University of Engineering, Handan, China; Universiti Sains Malaysia, MALAYSIA

## Abstract

Changes in precipitation patterns greatly impact regional drought/flood risk management and utilization of water resources. The main purpose of this paper was to investigate spatio-temporal variability of precipitation concentration in the transitional zone between Qinling Mountains (QDM), Guanzhong Plain (GZP) and the Loess Plateau (LPNS) in China, using monthly-scale precipitation concentration index (PCI) and daily-scale concentration index (CI) from daily rainfall records. The Mann-Kendall method was employed to illustrate the change in trend of PCI and CI, the Kriging interpolation method was adopted to measure spatial distribution, and the Wavelet transforms were used to explore their spatio-temporal correlation with the Arctic Oscillation (AO) & Western Pacific Subtropical High (WPSH) for revealing the potential attribution of precipitation concentration variation. Also, the regional implication of CI was investigated in the zone to provide local knowledge of the index application. Results showed that annual precipitation demonstrated a north-south increasing layered spatial distribution in the zone, representing a generally decreasing trend. The CI change generally exhibited a more significant decreasing trend than did PCI in LPNS and GZP due to AO slowly increasing over time, with a spatially weak layered or radial north-south decay, and an insignificant increasing trend in QDM impacted by the enhancing WPSH, with an obvious layered or radial spatial distribution. The spatiotemporal pattern of PCI variation represented similar characteristics in attribution with CI, but an inverse spatial distribution due to the phase difference (positive and negative effects) of AO and WPSH influencing seasonal precipitation. Regional analysis of CI showed that the CI value with over 0.62 indicated that approximately 80% of precipitation was contributed by 25% of the rainiest days in this zone. Fortunately, the area with this high CI has been getting smaller, implying a positive trend toward regional flash flood and debris flow control.

## Introduction

In recent years, the uneven spatial and temporal distribution of precipitation exacerbated by climate change has attracted much attention [1, 2]. The intensity [3], frequency [4], and pattern [5] of precipitation are expected to change, which can cause changes in evaporation and temperature [6], and more extreme weather events, such as floods [7] and droughts [8], are more likely to occur alternately. Meanwhile, numerous studies on precipitation variability, undertaken all over the world using various methods and indexes, indicate that wet periods had become longer over most of Europe [9] during 1950–2008, and a similar change in trend in wet periods occurred in Central America and northern South America [10] during 1961–2003. Differently, the wet periods were shortened in the Wei River basin during 1955–2012 [11] and precipitation had a negative trend with a 5% decline per century in Italy [12]. Sun et al. [13] found that the average daily rainfall intensity showed a significant regional declining trend in the Loess Plateau of China during 1960–2013, while the consecutive drought days showed a significant upward trend. Besides, several change points were found in the precipitation extremes in the Wei River basin, China [14], although there was no significant change in the annual precipitation. Recently, some researchers have argued that the changes in precipitation concentration represent seasonal characteristics of precipitation [15–17].

The variation of precipitation concentration is critical to water resource utilization, and it affects human life, environment, and ecosystem. For instance, the change of precipitation concentration is important to adjust the optimal allocation scheme of soil and water resources in time and to ensure higher yields of crops [18]. It is also needed for disaster prevention. The greater proportion of precipitation in rainy days to the total annual precipitation leads to a higher likelihood of floods. Intense rainstorms and floods trigger landslides [19], mudslides [20], and urban waterlogging [21]. The transitional zone between the Qinling Mountains and Loess Plateau is a typical rainstorm-induced flood area in China [22]. Therefore, it is important to investigate the changes in precipitation concentration for avoiding the loss of life and property as well as for coping with potential changes in water resources.

To investigate into precipitation concentration, two statistical indexes, i.e. concentration index (CI) depending on daily precipitation data and precipitation concentration index (PCI) depending on monthly precipitation data, were developed to illustrate the varying weight of daily precipitation and seasonal variation of precipitation, respectively. Two indices have been widely used in various countries and regions for precipitation concentration analysis. For example, Coscarelli et al. [23] computed CI and PCI to explore the spatial patterns of precipitation concentration in the Calabria region of Italy. Zamani et al. [24] applied the Mann-Kendall test to analyze the changes of CI and PCI in the Jharkhand state of India, and showed that the PCI values had a decreasing trend in eastern and northeastern parts of the study area, while CI varied irregularly and daily heavy rains had occurred over the study area in the period of 1971 to 2011. Zhang et al. [25] calculated CI based on daily precipitation data of the Pearl River basin, China, to illustrate the change in daily precipitation concentration. Li et al. [26] estimated the spatial heterogeneity of PCI values in various areas of Gansu province, China. They indicated that monthly precipitation had a strong irregular distribution during the year; however, the daily precipitation distribution tended to be uniform during the rainy season. Using daily rainfall records from 774 stations during 1961–2017, Lu et al. [27] investigated the spatio-temporal variability of precipitation concentration over Mainland China, and mapped the distribution of PCI and CI and significant trends in eight geographical subregions. Most studies have, however, focused on trend identification but the geographical distribution of precipitation concentration indices and their relationships with climate and geomorphic units were hardly noted.

Studies have shown that atmospheric circulation indices are an important factor affecting precipitation variability. Givati et al. [28] argued that AO can explain the changes in airflow that drive precipitation trends in the Mediterranean basin. Qu et al. [29] found that the springtime AO possibly influenced precipitation along the East Asian rain belt during 1979–2014. Studies in China indicated that there was a significant correlation between precipitation indices and AO in the Chinese Tianshan Mountains in wintertime during 1961–2011 [30]. Mao et al. [31] found that AO had a significant positive correlation with the frequency of extreme precipitation events over China from January to February, 1954–2009. Moreover, Liu et al. [32] observed positive multi-scale relations between drought variability and AO in Shaanxi province. Using cross wavelet transforms to analyze the correlation between AO and drought, Huang et al. [33] found that AO was strongly related to the propagation time from meteorological to hydrological drought in Wei River Basin. Not only does AO affect precipitation, but Western Pacific Subtropical High (WPSH) can also do. Li et al. [34] suggested that the strengthening WPSH was one of the main causes of the significant increasing precipitation in northwest China after the mid-1980s. The interannual variation of WPSH intensity was important for precipitation change, especially, in summer in Shaanxi [35]. The above studies point to the importance of correlation between precipitation concentration and atmospheric circulation indices in the long and short term which can be done using wavelet transforms.

Using Shaanxi province as a regional representative of the transitional zone between Qinling Mountains and Loess Plateau, this study performed a spatio-temporal analysis of precipitation concentration over the Qinling mountains, Guanzhong plain, and Loess Plateau, based on CI and PCI during 1951–2012. The objectives of this study therefore were to (1) investigate the tendencies and spatial distribution of CI, PCI, and annual precipitation by the Mann-Kendall test and a mapping technique, and reveal patterns of change in precipitation concentration; (2) analyze the migration of the distribution of precipitation concentration indices over three geomorphic units, and discuss the evolution of precipitation concentration patterns; (3) perform statistical analysis between CI value and precipitation concentration, and explore the regional implication of CI values; (4) build the relationship between precipitation concentration indices and atmospheric circulation indices (AO and WPSH) at multi-scale through the wavelet method (including Cross Wavelet Transform, XWT and Wavelet Coherence, WTC); and (5) illustrate the potential attribution of spatial and temporal migration of precipitation concentration indices occurring in the transitional zone between Qinling Mountains and Loess Plateau. The study will provide insights into changes in precipitation concentration over various geomorphic units, regional implications of CI values and the influence of atmospheric circulation indices, which are beneficial for understanding precipitation patterns under changing climate and potential risks from flood or drought disasters in the region.

## Materials and methods

### Study area and data

**Fig 1:**
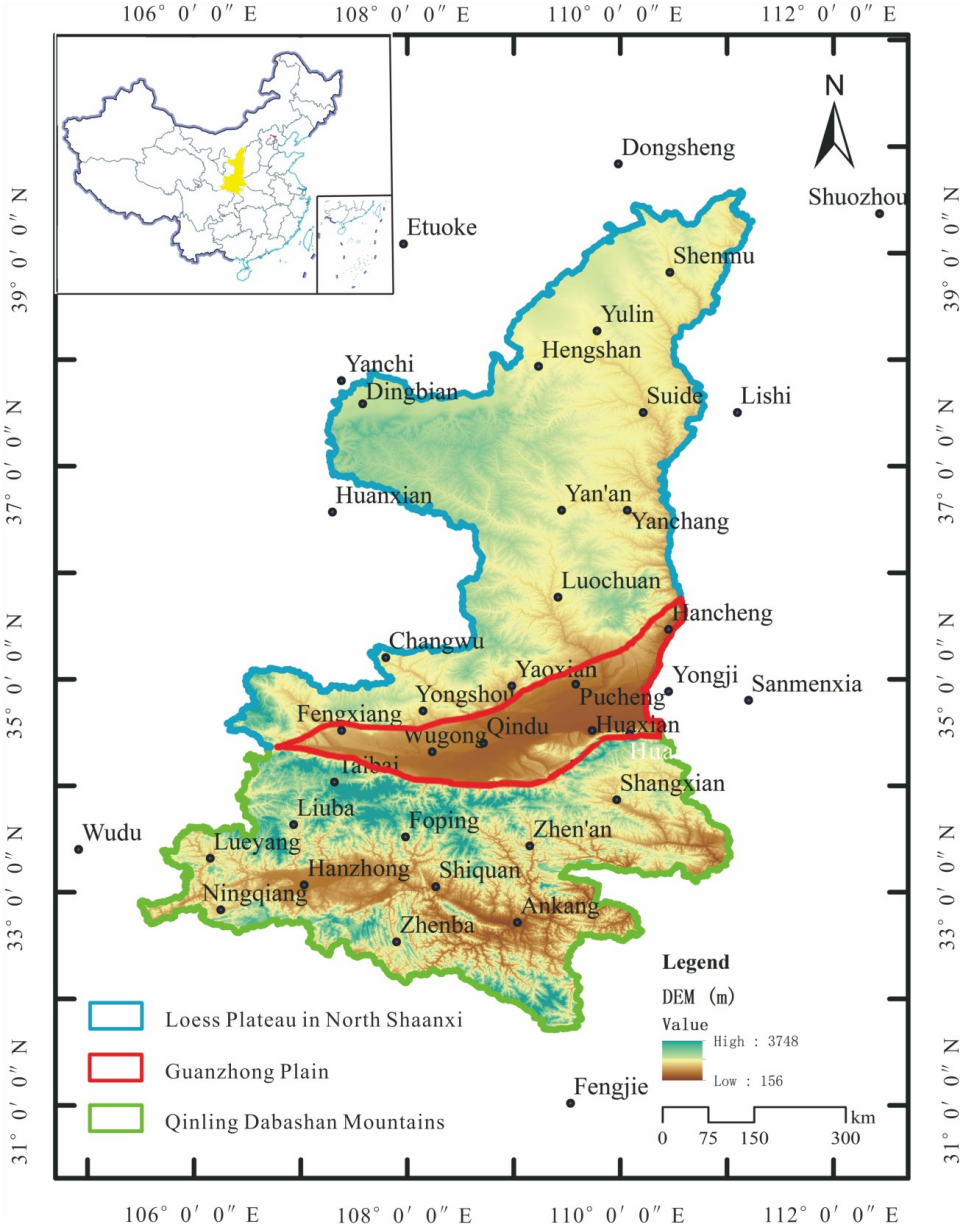
Location of the study area with 39 selected meteorological stations.

Shaanxi Province is located in the east of northwestern China (Fig 1), ranging from 31°42′ to 39°35′N and from 105°29′ to 111°15′E [36]. It covers an area of 205, 800 km^2^, with an altitude range from 300 m to 3500 m. This province lies in the transitional zone between Qinling Mountains and Loess Plateau from climate varying from humid to arid [37], covering three geomorphic units (as shown in Fig 1): Qinling Dabashan Mountains (QDM), Guanzhong Plain (GZP), and Loess Plateau in North Shaanxi (LPNS). Correspondingly, Shaanxi province consists of three climatic zones from south to north: subtropical humid zone, warm temperate semi-humid zone, and warm temperate semi-arid zone. It leads to a wide variation of precipitation pattern in space. In general, Shaanxi province is a typical case sensitive to climate variation, with mean annual temperature of 13°C and average annual rainfall of 580 mm. Most of the annual precipitation falls in May-October [38]. Influenced by the spatial heterogeneity of precipitation, it is, therefore, particularly important to investigate the characteristics of changes in precipitation concentration in time and space in Shaanxi province or the transitional zone under changing climate (natural and human-induced).

In this study, daily rainfall data records from 39 meteorological stations (Fig 1) across and around the study area were collected from the China Meteorological Data Sharing Service System (available at http://data.cma.cn/data/cdcdetail/dataCode/SURF_CLI_CHN_MUL_DAY_V3.0.html), and monthly and annual precipitation series were calculated based on daily data. Details of 39 stations are shown in Table 1, of which 29 stations are located in the study area (including 11 in LPNS, 7 in GZP, 11 in QDM), and 10 stations outside of the study area. The data outside are mainly used for spatial mapping of CI from daily precipitation and PCI from monthly precipitation.

**Table 1:** Details of 39 selected meteorological stations.

Name	Location	Data	Name	Location	Data	Name	Location	Data
**Shenmu**	LPNS	1957–2012	**Qindu**	GZP	1960–2012	**Ankang**	QDM	1953–2012
**Yulin**	LPNS	1951–2012	**Pucheng**	GZP	1959–2012	**Zhen’an**	QDM	1958–2012
**Hengshan**	LPNS	1954–2012	**Huaxian**	GZP	1961–2012	**Shangxian**	QDM	1954–2012
**Dingbian**	LPNS	1957–2012	**Hancheng**	GZP	1957–2012	**Sanmenxia**	Outside	1957–2012
**Suide**	LPNS	1953–2012	**Huashan**	GZP	1953–2012	**Yongji**	Outside	1972–2012
**Yan‘an**	LPNS	1951–2012	**Taibai**	QDM	1957–2012	**Shuozhou**	Outside	1957–2012
**Yanchang**	LPNS	1957–2012	**Liuba**	QDM	1958–2012	**Lishi**	Outside	1957–2012
**Luochuan**	LPNS	1955–2012	**Lueyang**	QDM	1954–2012	**Fengjie**	Outside	1954–2012
**Changwu**	LPNS	1957–2012	**Ningqiang**	QDM	1957–2012	**Wudu**	Outside	1951–2012
**Yongshou**	LPNS	1959–2012	**Hanzhong**	QDM	1951–2012	**Dongsheng**	Outside	1957–2012
**Yaoxian**	LPNS	1959–2012	**Zhenba**	QDM	1959–2012	**Etuoke**	Outside	1954–2012
**Fengxiang**	GZP	1959–2012	**Shiquan**	QDM	1960–2012	**Huanxian**	Outside	1957–2012
**Wugong**	GZP	1955–2012	**Foping**	QDM	1957–2012	**Yanchi**	Outside	1954–2012

### Methods

#### Concentration index

Concentration index (CI), proposed by Martin-Vide [39], is an index that represents the concentration of daily precipitation. It mainly assesses the relative impacts of different magnitudes or classes of daily precipitation and evaluates the weight of the days recording the largest daily rainfall event [40, 41]. In this study, a day with rainfall exceeding 0.1 mm was regarded as the rainy day, and 1 mm interval was used to classify rainfall events. The calculation steps were as follows: (1) determining the rainfall limits of *i*th classes (*i* = 1,2,…, *M*); (2) counting the number of days with precipitation falling in each class, *N*_*i*_; (3) calculating the cumulative amount of daily precipitation in each class, *P*_*i*_; (4) summing the cumulative values from Steps (2) and (3), and obtaining ∑*N* and ∑*P*; (5) computing the accumulated percentages of rainy days (∑*N*_*i*_(%) or *X*_*i*_) and the associative accumulated percentages of precipitation (∑*P*_*i*_ (%) or *Y*_*i*_) responding to *i*th class; and (6) drawing the exponential curve of *X* versus *Y*. This curve is named as concentration curve or Lorenz Curve, and has been applied in many countries and regions worldwide [25, 27, 36, 42, 43].

There the pairs of value (*X*_*i*_, *Y*_*i*_) were defined as

$$Y=a\times X\times e^{bX}$$ (1)

where *a* and *b* are the regression coefficients that can be determined by the least-squares method as follows:

$$\text{ln}\;a=\frac{\sum X_{i}^{2}\sum \text{ln}\;Y_{i}+\sum X_{i}\sum X_{i}\;\text{ln}\;X_{i}-\sum X_{i}^{2}\sum \text{ln}\;X_{i}-\sum X_{i}\sum X_{i}\;\text{ln}\;Y}{M\sum X_{i}^{2}-{\left(\sum X_{i}\right)}^{2}}$$ (2)

$$b=\frac{M\sum X_{i}\text{ln}\;Y_{i}+\sum X_{i}\sum \text{ln}\;X_{i}-N\sum X_{i}\;\text{ln}\;X_{i}-\sum X_{i}\;\text{ln}\;Y_{i}}{M\sum X_{i}^{2}-{\left(\sum X_{i}\right)}^{2}}$$ (3)

**Fig 2:**
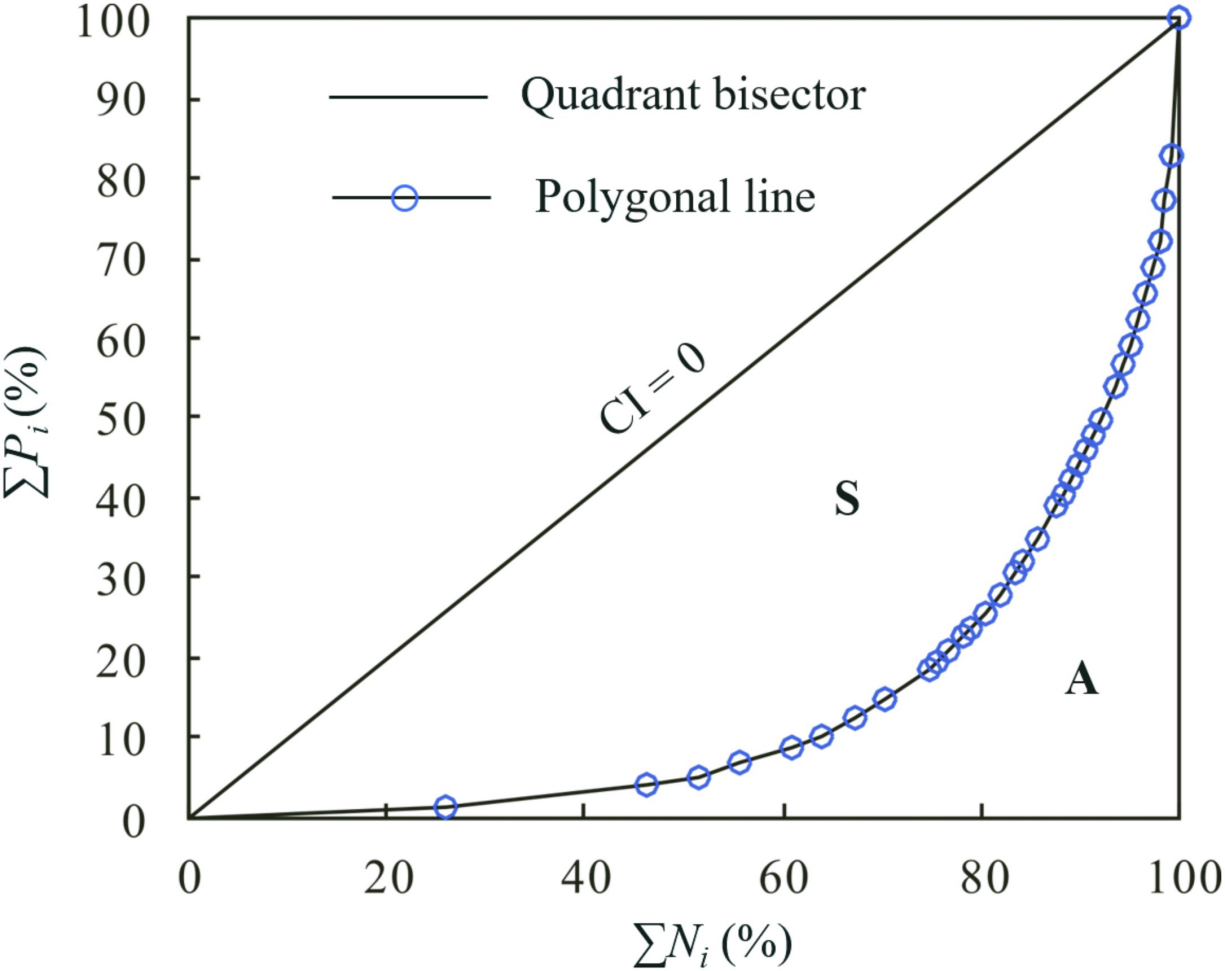
Concentration or Lorenz curves for daily precipitation.

The daily precipitation concentration index (CI) was defined as

$$CI=\frac{S}{5000}$$ (4)

$$S=\frac{10000}{2}-A$$ (5)

$$A=\int_{0}^{100}\frac{a}{b}e^{bx}(x-\frac{1}{b})dx$$ (6)

where *S* is the area, which is enclosed by the polygonal line and the bisector of the quadrant (equidistribution line), and *A* is the area under the curve, as shown in Fig 2.

The CI value is the proportion of *S* in the lower triangle, which is defined by the equidistribution line, ranging from 0 to 1. The bigger the area of *S*, the larger the CI value, indicating the higher the concentration.

#### Precipitation concentration index

Precipitation concentration index (PCI), defined by Oliver [44], is also a powerful indicator for temporal precipitation distribution. Similar to CI, PCI is generally used for evaluating seasonal precipitation changes to investigate the heterogeneity of monthly rainfall data. The calculation is described as follows:

$$PCI=100\times \frac{\sum_{i=1}^{12}p_{i}^{2}}{{\left(\sum_{i=1}^{12}p_{i}\right)}^{2}}$$ (7)

where *p*_*i*_ is the rainfall amount of month *i*. According to Eq (7), PCI explains the degree of precipitation concentration during a given year on an annual scale, ranging from 8.3 to 100. It is suggested by Oliver [44] that when the PCI value is less than 10, the precipitation is uniformly distributed throughout the year, and when the PCI value is from 10 to 15, precipitation is moderately concentrated, and if the values vary from 16 to 20, the precipitation has an irregular distribution, and the precipitation with PCI over 20 represents a significant irregular distribution [45].

#### Mann–Kendall test

The non-parametric Mann-Kendall method (MK), proposed by Mann et al. [46] and Kendall et al. [47], is widely employed to detect monotonic trends in the time series of hydrometeorological variables, including temperature [48], streamflow [49], and rainfall [50]. This method does not require that the detected data adhere to a specific distribution and is disturbed by other parameters. In the calculation, the value of Z is the judgment criterion for the trend change [51, 52]. When |*Z*| ≤ 1.96, the null hypothesis *H*_0_ is accepted, indicating that there is no significant trend at the 0.05 significance level. |*Z*| ≥ 1.96 demonstrates the trend of the time series is statistically significant. It must be noted that a positive *Z* indicates that the sequence has an increasing trend, while a negative *Z* reflects a declining trend.

#### Wavelet transforms

Wavelet transforms were adopted in this study, including Cross Wavelet Transform (XWT) [53] and Wavelet Coherence (WTC) [54], aiming to reveal information about phase relationships between precipitation and atmospheric circulation indices. Therein, XWT reveals areas with high common power, and WTC reveals the coherence of XWT in time-frequency space [55]. As another useful measure, WTC makes up for the lack of correlation analysis with low common power in XWT [56]. These two analysis approaches are always used in a complementary way [57].

At present, XWT is generally used to analyze the change characteristics and coupled oscillations of the two series, X and Y, both in time and frequency domains [58]. The cross-wavelet spectrum of X and Y is defined as:

$$W{\left(k\right)}^{XY}=W{\left(k\right)}^{X}W{\left(k\right)}^{Y^{*}}$$ (8)

where * denotes complex conjugation. *W* is the wavelet transform of X and Y at the wavelet scale *k*. The cross wavelet power is expressed as |*W*(*k*)^*XY*^|, and the phase angle of *W*(*k*)^*XY*^ notes the local relative phase relationship between X and Y in the time-frequency space. The statistical significance of the wavelet power is estimated against a red noise model.

The wavelet coherence, different from the cross-wavelet, is defined as:

$$R_{n}^{2}(k)=\frac{{\left|m(k^{-1}W^{XY}(k))\right|}^{2}}{m(k^{-1}{\left|W^{X}(k)\right|}^{2})\cdot k(k^{-1}{\left|W^{Y}(k)\right|}^{2})}$$ (9)

where *m* is a smoothing operator to help think of the wavelet coherence as a localized correlation coefficient in time frequency space. Monte Carlo method is used to estimate the significance level of the wavelet coherence.

## Results

### Space-time changes in annual precipitation

**Fig 3:**
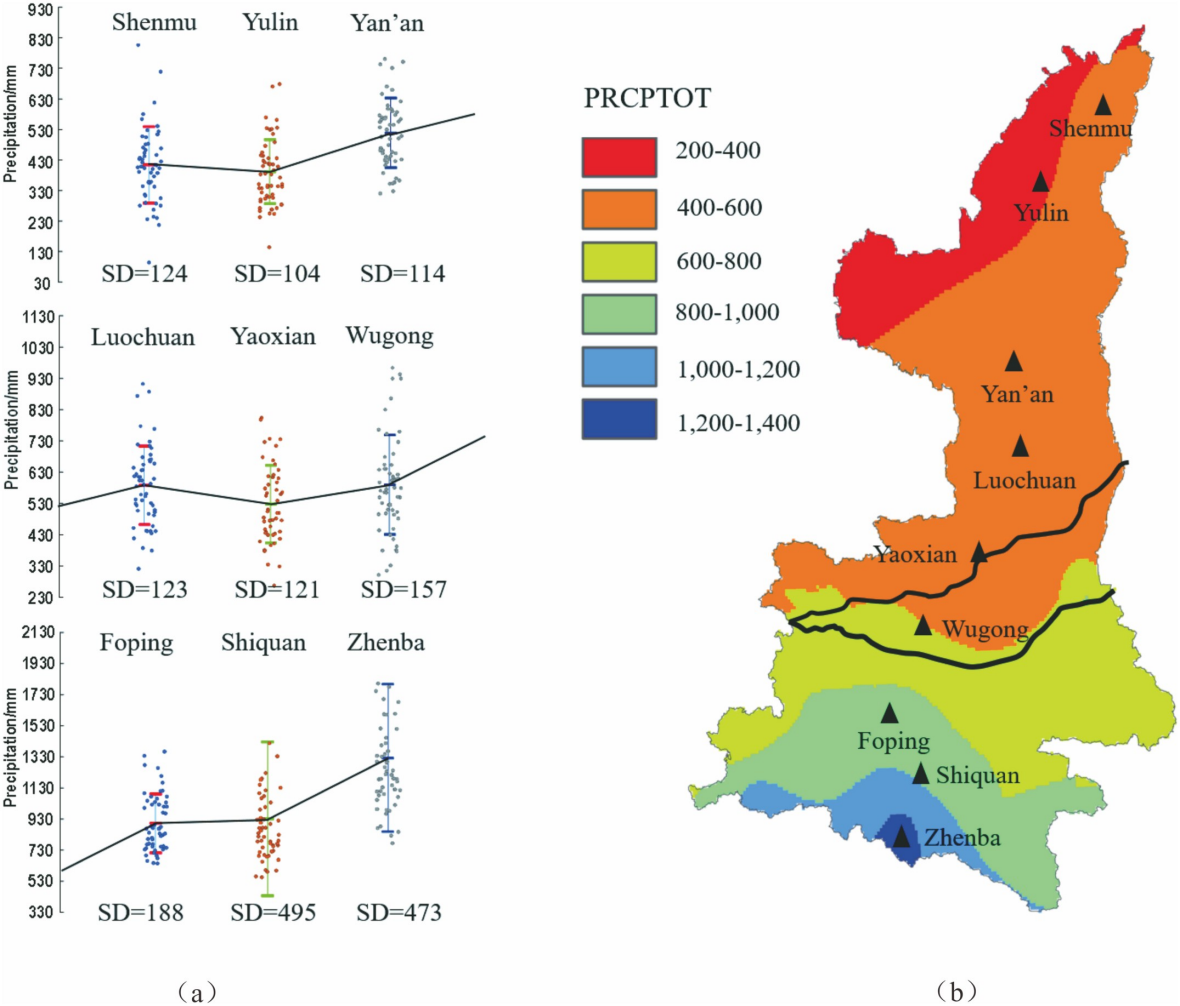
Statistical analysis of interannual average precipitation from 8 stations. SD notes the standard deviation squared (variance), and PRCPTOT notes the total annual precipitation.

Statistical analysis of interannual average precipitation from 29 stations inside the study area was done, depending on the annual rainfall data. Fig 3a shows the statistical results of precipitation at nine stations (i.e. Shenmu, Yulin, Yan’an, Luochuan, Yaoxian, Wugong, Foping, Shiquan, and Zhenba) across the transitional zone from Qinling Dabashan Mountains (QDM) to Guanzhong Plain (GZP) to Loess Plateau in North Shaanxi (LPSN). It can be seen from the figure that interannual average precipitation had an increasing trend from north to south in Shaanxi Province, ranging from 367 mm to 1322 mm, and the variance of annual precipitation series rose gradually.

Fig 3b maps the spatial pattern of precipitation in the study area, indicating that precipitation demonstrates an obvious layered distribution in the whole region. The low precipitation of 200–400 mm mainly fell in the Mu US Desert in Northwest Shaanxi, and the medium precipitation ranging from 400 to 800 mm covered most of LPNS, GZP, and the north foot of QDM, and the high precipitation of more than 800 mm occurred in the south area of QDM. From the spatial variation of precipitation, QDM is an area with strong variation in precipitation, representing an average growth of about 3–4 mm/km along the spatial gradient of precipitation in the south Mountain area.

**Fig 4:**
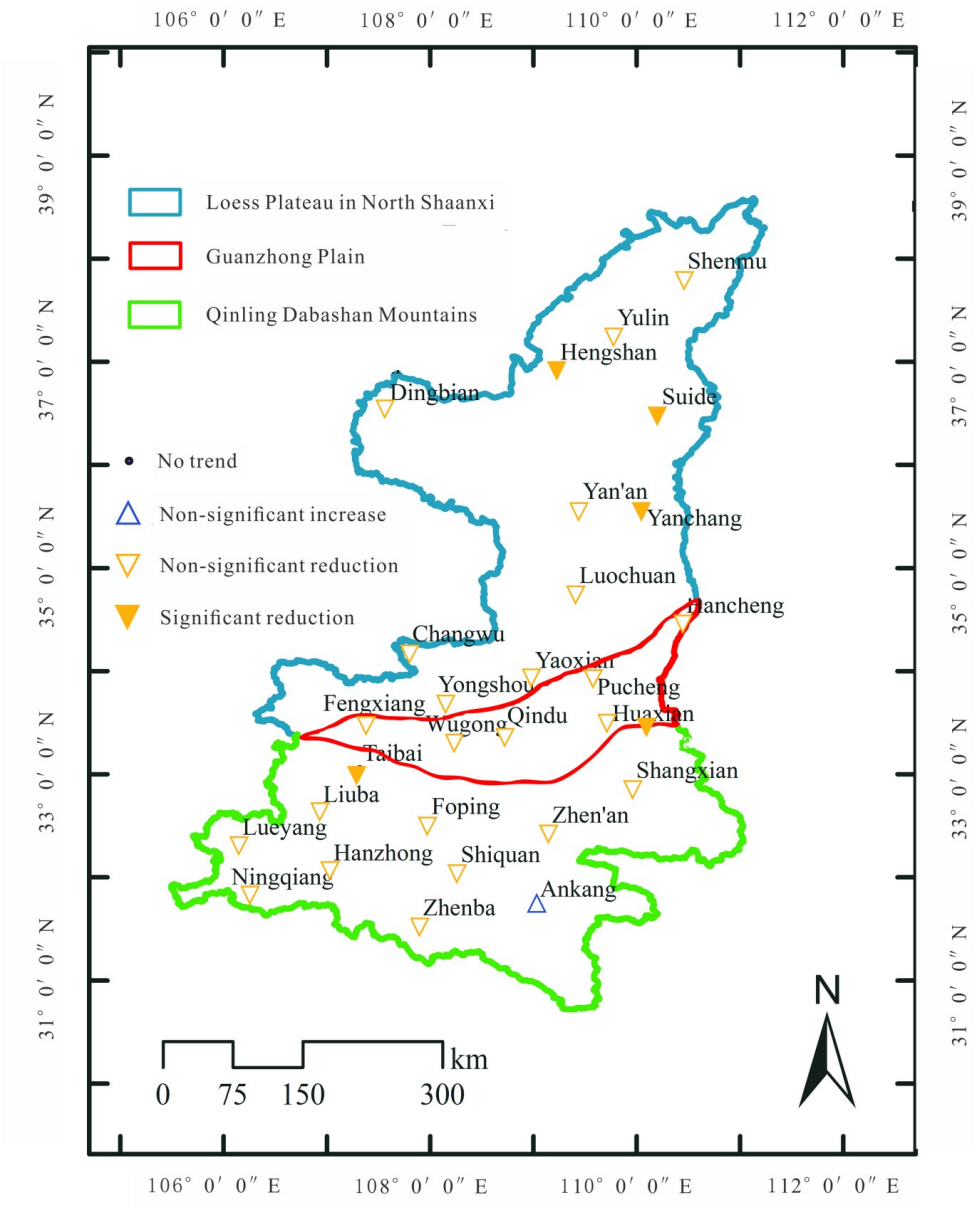
MK test results of annual precipitation from 29 stations in the study area.

Also, the Mann-Kendall test was used to investigate changes in the trend of annual average precipitation from 29 internal stations. As shown in Fig 4, results demonstrated that the annual precipitation from all stations (except Ankang in the central QDM) showed decreasing trends. Therein, Hengshan, Yanchang, Suide stations in PLNS; Huashan station in GZP; and Taibai station in the west QDM represented significant declining precipitation. Also, the Z index from 10 stations was more than 1, including Shenmu, Yulin, Yan’an stations in LPNS; Yongshou station at the junction of LPNS and GZP; Huaxian, Pucheng, Wugong station in GZP; and Liuba, Ningqiang, and Hanzhong in western QDM, implying that the decreasing precipitation basically covered the whole study area (except east-central QDM).

The stations in LPNS with declining precipitation were located in the main population areas and economic development areas. The annual precipitation change would pose a great challenge to regional water security and ecological protection, because water scarcity has been severe in LPNS. Meanwhile, the western QDM is in the drainage area of the Huangjinxia reservoir which is the primary water resources area for the Hanjiang to Wei River Water Diversion Project under construction. The task of the project is to transfer an average of 1.5 billion m^3^ water per year from the Han River to the Guanzhong area of Shaanxi Province, serving important cities, counties, and industrial parks [59]. Unfortunately, precipitation in the GZP as the intake area of the project has been declining. One can then infer that the diversion project would be risky, and could cause the financial loss of the intake area when precipitation in the primary water resources area and intake area would decrease significantly.

In addition, it is noted that the concentration variations of precipitation (such as PCI and CI) as well as streamflow in the diversion and intake areas should be paid more attention in order to ensure the realization of the project goal. Only when the changes in precipitation concentration are limited, the diversion project transferring the water from the Han River in the wet period to the intake area in the Wei River basin would work well and would provide enough water for use in dry periods in the diversion and intake areas.

### Spatial patterns of precipitation concentration indices

**Fig 5:**
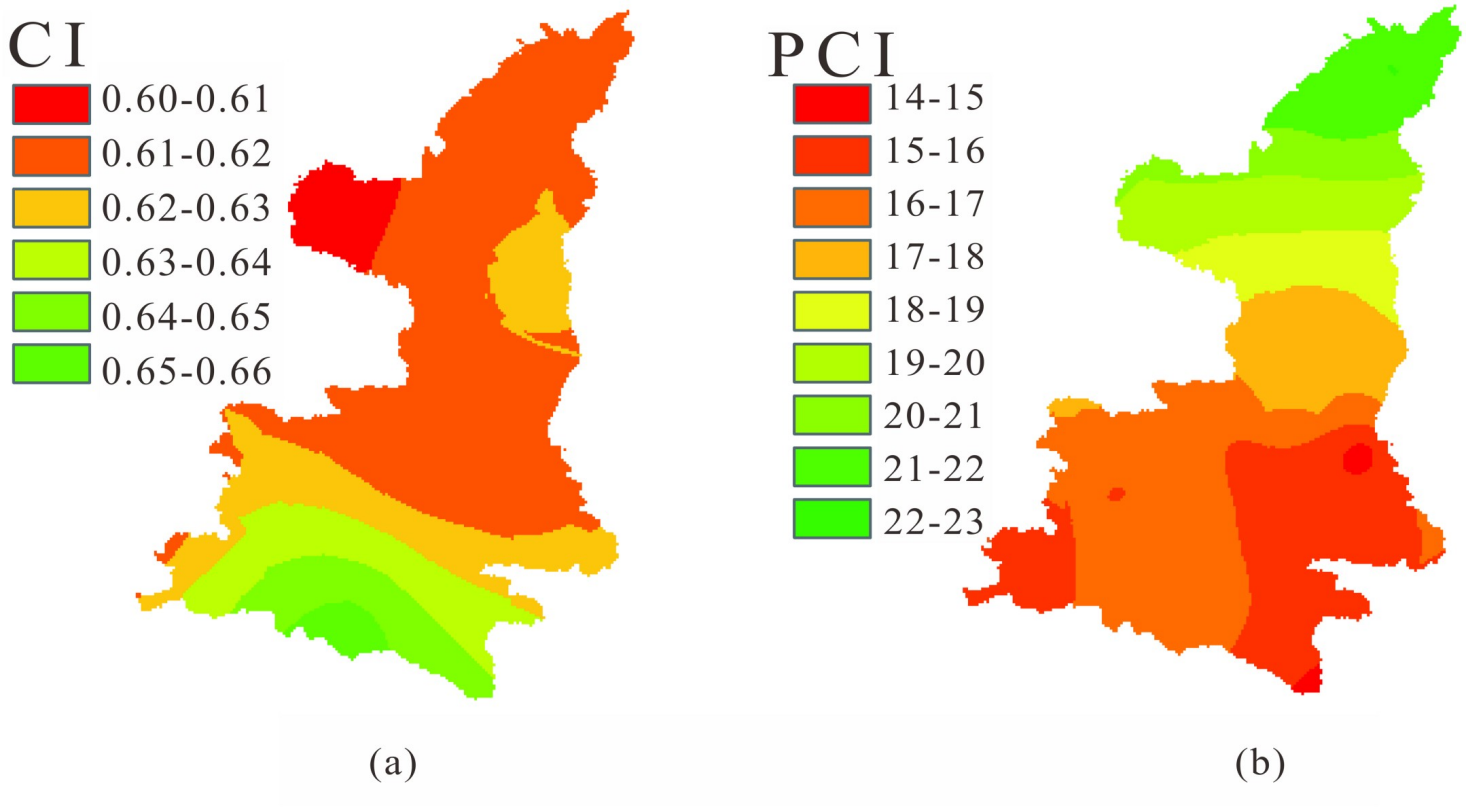
Spatiotemporal patterns of CI (a) and PCI (b) in Shaanxi province.

The spatial distributions of interannual average CI and PCI values from 39 meteorological stations were analyzed in and around the study area, and the mapping results are shown in Fig 5. To map the precipitation concentration indices, the Kriging interpolation method was adopted to illustrate the spatial distribution.

From Fig 5a, it is observed that the CI values, with slight variations ranging from 0.6 to 0.66, exhibited a decreasing trend from south to north in Shaanxi Province. Therein, the CI values from Dingbian station and its vicinity were minimum, and the area around Zhenba station had the maximum CI. It should be noted that the figure demonstrated the obvious layered distribution in south Shaanxi, and the phenomenon was considered related to the climate indices influencing the region from the south.

The PCI values mainly varied from 14 to 23, shown in Fig 5b, illustrating a rising trend from the south region to the north region, contrary to the spatial distribution of CI. It was found from the figure that the lowest PCI values appeared near the Huashan station and the southeastern corner of Shaanxi province, less than 15, representing the moderately uniform seasonal variation of precipitation in the region according to Oliver [44]. The PCI values in other parts of central and southern Shaanxi were between 16 and 20, demonstrating that the monthly precipitation distribution was irregular, i.e. most precipitation in the wet period of June-October in the area. In most of the northern region, PCI value exceeded 20, indicating significant irregular precipitation distribution throughout the year. It represents that more than 70% of rainfall was falling in just three months of July-September in this region. Similarly, the obvious layered distribution occurred in the PCI mapping, mainly focusing on the LPNS, and it should be also relevant to the climate oscillation from the north.

Comparing the spatial distributions of two indicators, it can be found that the CI shows an opposite pattern of PCI in Shaanxi province. Considering the obvious layered distribution and the occurring location in the mapping of CI and PCI, we can infer that there are different climate oscillations influencing the spatial distribution and temporal change of CI and PCI, respectively or together. However, the current results do not support the analysis of the attribution. Therefore, the issue will be further discussed based on potential atmospheric circulation indexes in later sections.

### Temporal patterns of precipitation concentration indices

**Fig 6:**
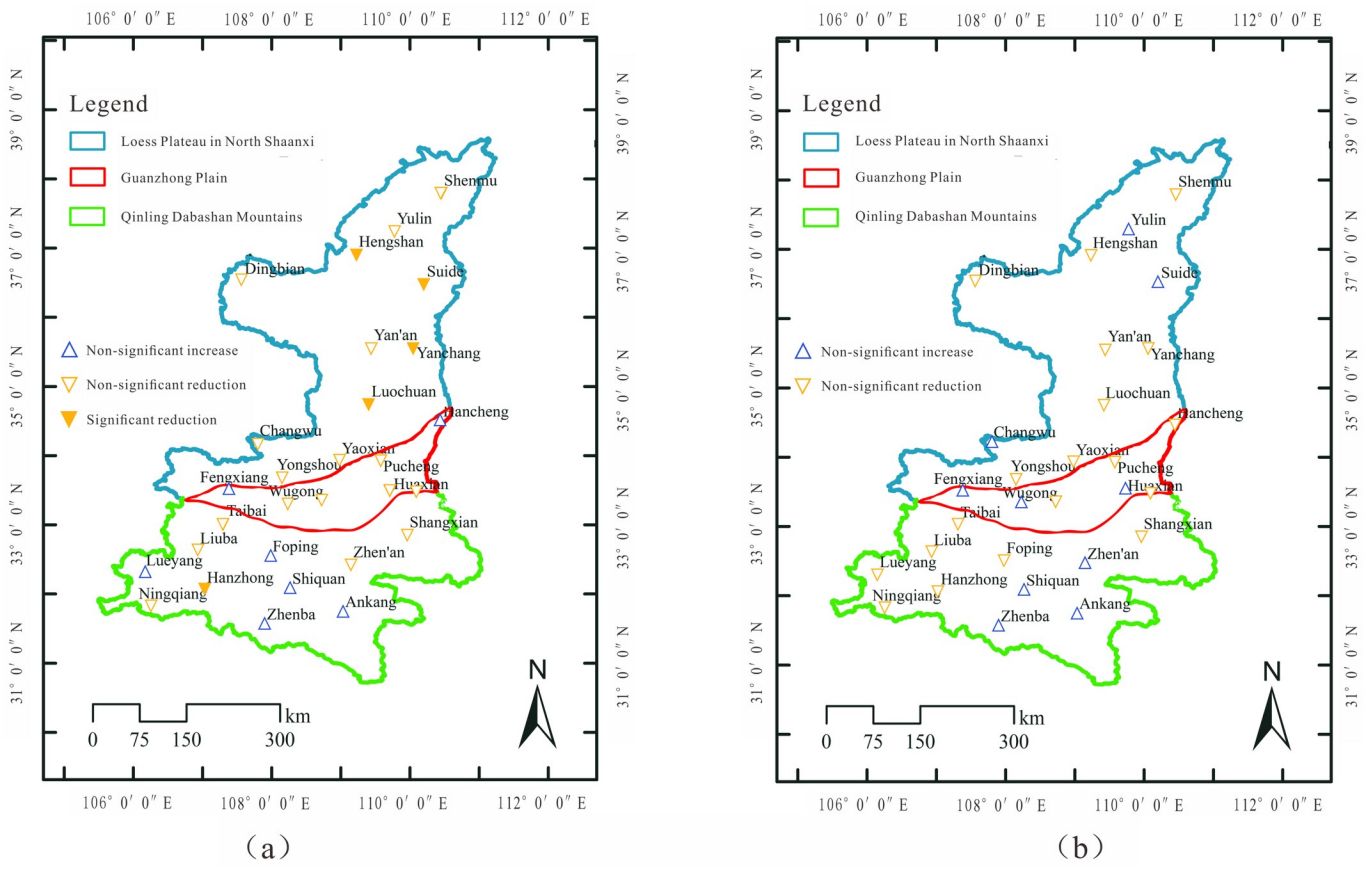
MK test results of CI (a) and PCI (b) at 29 stations in the study area. The trend significance is under 95% confidence interval.

The Mann-Kendall (MK) test was employed to implement the detection of change in the trend of CI and PCI series, for providing an insight into temporal patterns of precipitation concentration. Results of CI, as shown in Fig 6a, demonstrated that CI from all stations showed a decreasing trend in the past 60 years, except from two stations at the junction of PLNS and GZP (i.e. Fengxiang and Hancheng) and five stations in the western & central QDM (i.e. Lueyang, Foping, Shiquan, Ankang, and Zhenba). Therein, Hengshan, Suide, Yanchang, and Luochuan stations in PLNS and Hanzhong stations in the western QDM had significant declining precipitation. The decreasing CI in the study area is helpful for flash flood and debris flow control, while the increase of CI in the central QDM implies that large floods could be more frequent than ever, although the rise is not significant.

Different from the change in trend of CI, the PCI from 29 stations in the study area didn’t represent a significant change in trend (Fig 6b). A third of all stations showed increasing PCI, distributed in northern LPNS, the western GZP and central QDM. The PCI values from the rest of stations were with a downward trend. Combining the changes of annual precipitation and PCI in the western QDM as the primary water resources area of the Hanjiang to Wei River Water Diversion Project, a point is emphasized that when regional annual precipitation and PCI would both decrease greatly in the future, the water from the wet period can be diverted outside of the area, which could be less than that is expected in the project design. Thus, it is suggested that the water available in the wet period for diversion should be recalculated, considering the PCI change and the risk should be reevaluated.

Comparing Fig 6a and 6b, it can be found that PCI and CI were all rising in the statistical period at Zhenba, Ankang, Shiquan stations in central QDM and at Fengxiang station in the western GZP, although annual precipitation at all stations (except Ankang) was reducing. The phenomenon illustrated that regional precipitation was very concentrated at daily and monthly scales, prone to frequent natural disasters, such as floods. On the contrary, the reduction of PCI and CI in PLNS, GZP and western QDM indicated that precipitation would be more uniform in these regions, implying that the occurrence of high-magnitude flash flood/debris flow events and the challenge of water resources utilization were decreasing. Of course, the decrease of annual precipitation in these areas was still a barrier against sustainable regional water resources development, and would still impact water and ecological security in these areas.

**Fig 7:**
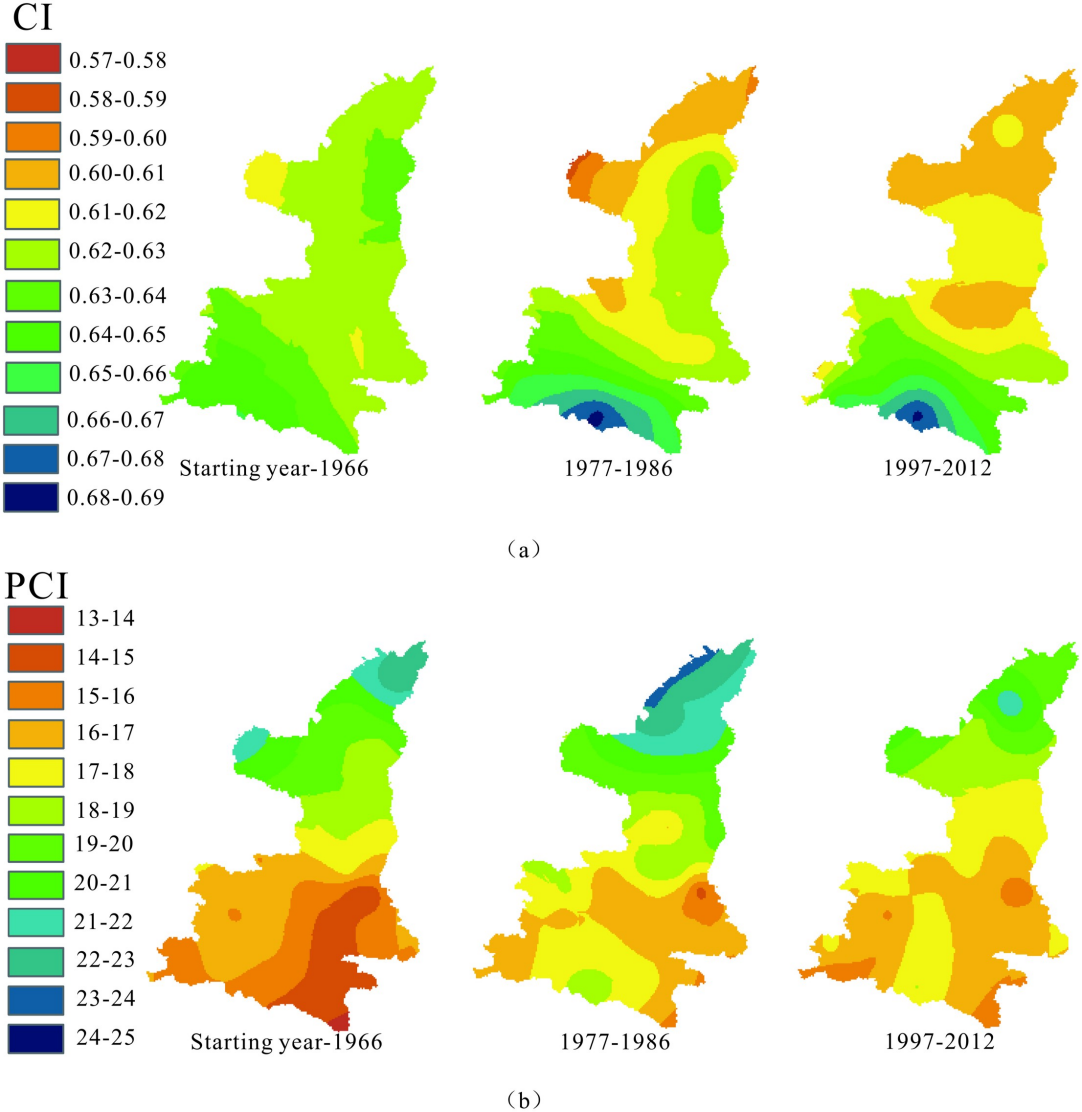
Spatial patterns of CI (a) and PCI (b) in Shaanxi province in three periods.

To investigate the temporal evolution of the distributions of CI and PCI, the mappings of CI and PCI during three periods (starting year-1966, 1977–1986, 1997–2012) were constructed, as shown in Fig 7. It is seen from Fig 7a that the range of CI change in the study area increased over time, representing the intervals of 0.58–0.69 in 1977–1986 and 0.60–0.69 in 1997–2012, larger than the interval of 0.61–0.65 before 1966. It revealed that CI in southern Shaanxi exhibited an increasing trend and that in northern Shaanxi had a decreasing trend after 1977, and this change was gradual but obvious over time. Up to 2012, the area with CI less than 0.61 had accounted for 32.49% of the whole study area, while the area was zero before 1966. Also, the phenomenon of layered spatial distribution of CI strengthened over time, and the horizontally layered characteristics had been greatly obvious during 1997–2012. Especially in southern Shaanxi, the spatial distribution of CI represented concentric semi-circles around Zhenba station, different from which the concentric circle didn’t occur in northern Shaanxi, and the horizontal layered distribution mainly occurred. In our opinion, the change of CI’s spatial distribution overtime was not a simple decay with distance influenced by atmospheric circulation indices coming from south, but was synthetically impacted by atmospheric circulation indices from south and north, especially during 1997–2012.

The range of PCI spatial distribution (Fig 7b) showed a weak decreasing trend from 13–23 before 1966 to 14–25 in 1977–1986, and to 15–22 during 1997–2012. In these three times, the PCI of most study areas concentrated in 16–19, covering about 41.4%, 63.8% and 76.51% of the study area before 1966, in 1977–1986, and 1997–2012, respectively. Also, the PCI was layered indistinctively, representing the decrease from north to central Shaanxi. Similarly, the change over time should be relevant to atmospheric circulation indices, and the detail will be probed in the discussion section.

## Discussion

Although the spatiotemporal patterns of CI and PCI in Shaanxi Province have been explored, two issues need further investigation, i.e. the attribution of the temporal evolution of spatial distribution of CI and PCI and regional implication of these precipitation concentration index values.

### Potential impact of atmospheric circulation indices

As mentioned in the above section, the temporal evolution of CI and PCI could be related to atmospheric circulation indices, representing that changes in the strength of atmospheric circulation indices would impact the spatial distribution of precipitation concentration indices over time. To explore this issue, a series of atmospheric circulation indices were first used to build their relationship with monthly precipitation at representative stations in Shaanxi Province, aiming to provide insights about the attribution of the temporal evolution of precipitation concentration indices. Finally, Arctic Oscillation (AO) and Western Pacific Subtropical High (WPSH) were found relevant with precipitation in different months in Shaanxi Province, respectively. A similar phenomenon had been found in Northern China [60].

**Fig 8:**
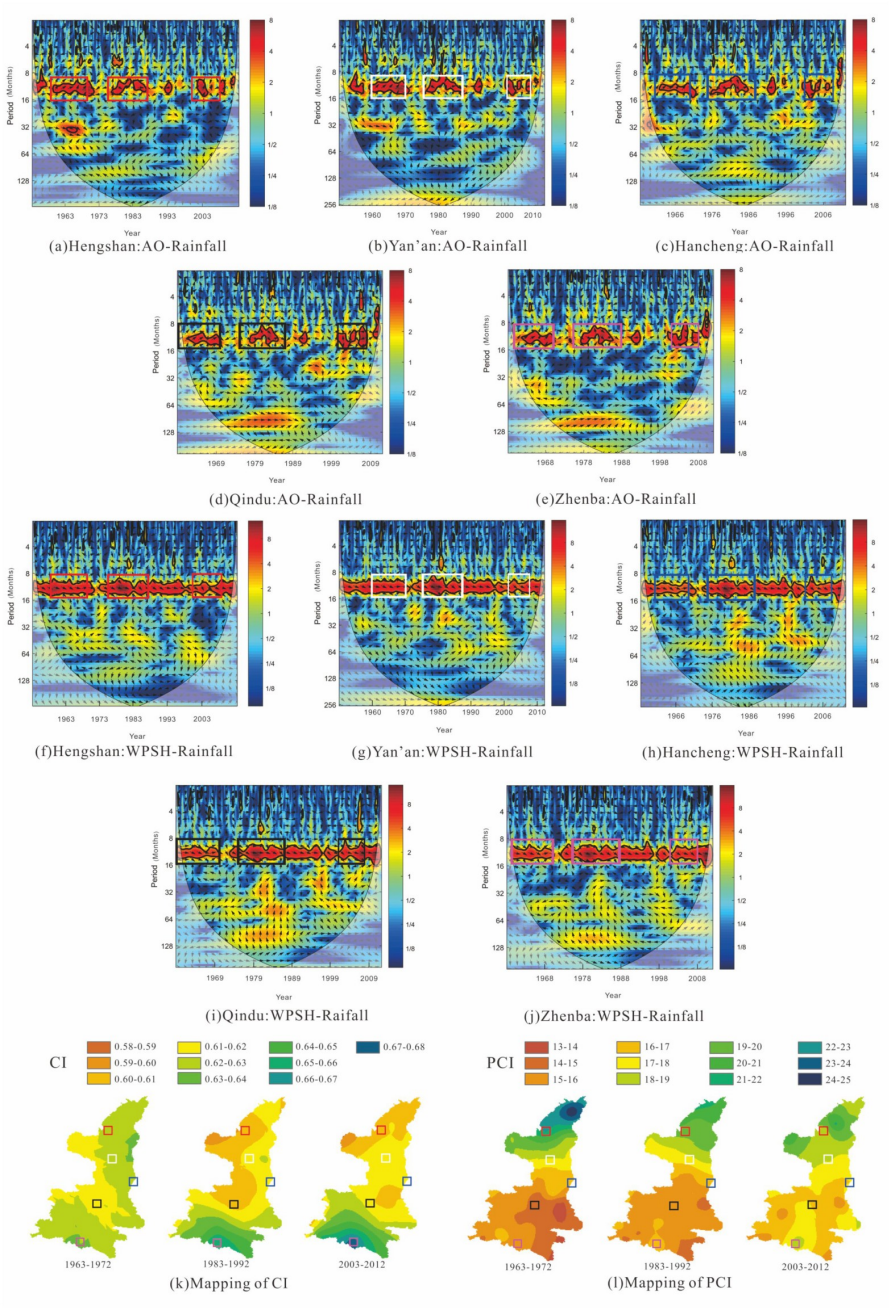
Cross-wavelet transform of monthly AO & WPSH and monthly rainfall at Hengshan (a, f), Yan’an (b, g), Hancheng (c, h), Qindu (d, i), and Zhenba (e, j) stations as well as the mappings of CI (k) and PCI (l) in three periods. In subfigures (a)-(j), the thick solid line is the 5% significance level, and the thin solid line indicates the cone of influence.

To clarify the relationship between changes of CI and PCI and two atmospheric circulation indices, the cross-wavelet transform (XWT) and wavelet coherence (WTC) of monthly precipitation and monthly AO & WPSH were performed to reveal the pattern of influence of atmospheric circulation indices on precipitation. Cross-wavelet transform of AO-rainfall and WPSH-rainfall at the monthly scale are shown in Fig 8, including XWT of AO and WPSH with monthly precipitation at Hengshan and Yan’an stations in PLNS, Hancheng and Qindu stations in GZP, and Zhenba station in QDM.

From Fig 8a–8e, it can be found that XWT between monthly AO and precipitation indicated common features in the wavelet power. They represented that the significant correlation at 12-month scale occurred in 1960–1970, 1975–1988, and 2001–2008, and that AO had a high influence on monthly precipitation. Similarly, XWT between monthly WPSH and precipitation, as shown in Fig 8f–8j, demonstrated stronger common features at the 12-month scale, and had higher wavelet power than AO-rainfall over time, implying a significant effect on monthly precipitation changes.

Comparing with temporal changes of PCI in three periods (before 1966, 1977–1986, 1997–2012), it is seen from XWT of AO-rainfall that PCI changes at five stations had a similarity with that of the significant area with common power, showing that the PCI value was the largest in 1977–1986 with the highest wavelet power, and PCI was the lowest before 1966 with the smallest significant area in the figures. It indicates that the influence of AO on monthly precipitation would further affect the monthly precipitation concentration, PCI. Although WPSH had a more significant relationship with monthly precipitation at the five stations, the impact on PCI was not obvious, representing that the XWT of WPSH-rainfall failed to match the changes of PCI in different periods. Focusing on the relationship of CI and the XWT of WPSH-rainfall and AO-rainfall, it was observed that the changes of AO and WPSH had no obvious relation with that of CI, illustrating that the influence of AO & WPSH on the daily precipitation concentration (CI) was not well deciphered by the analysis at the current scale.

**Fig 9:**
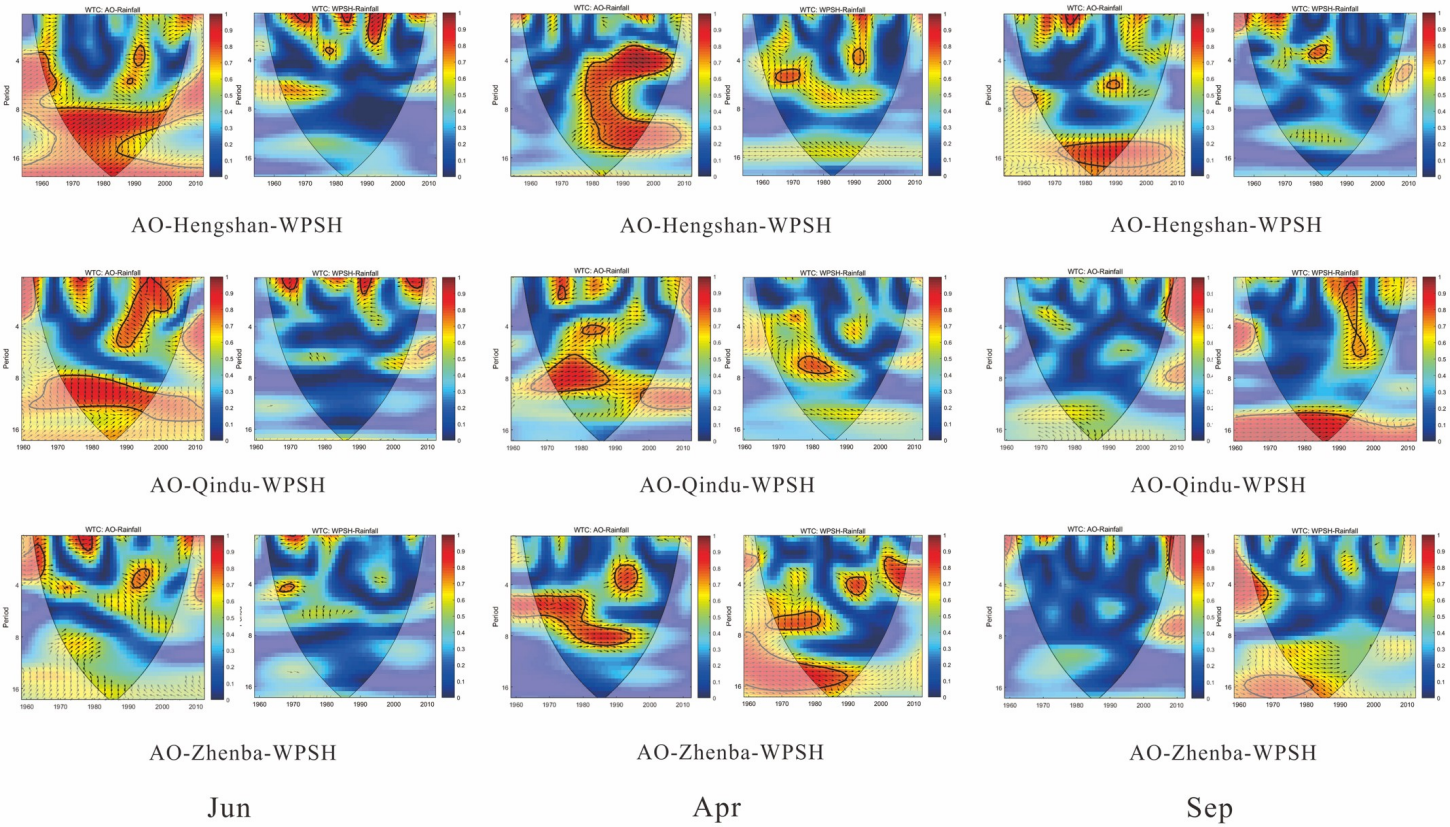
Wavelet coherence of monthly AO & WPSH and monthly rainfall at Hengshan, Qindu and Zhenba stations.

To investigate the impact of AO and WPSH on monthly precipitation, the WTCs of AO-rainfall and WPSH-rainfall in twelve months were obtained. Results indicated that the WTC of AO-rainfall was highly significant in Jan, Apr, May, Sep, and Nov, and WPSH had a significant correlation with rainfall in Feb, Mar, Apr, Sep and Nov. The representative results at Hengshan, Qindu, and Zhenba stations are shown in Fig 9, including WTC in Jan (dry season), Apr (before flood season), and Sep (flood season).

From WTC of AO-rainfall and WPSH-rainfall, it can be observed that AO mainly affected monthly rainfall in the dry season and before flood season in the in-phase, representing that greater the AO index, higher the precipitation, and had a weak chaotic-phase relationship with rainfall in the flood season in a year. Similarly, WPSH greatly impacted precipitation in the non-flood season, however, its influence was varying in several months with a significant impact, showing different phases over time in WTC, as shown in Fig 9. It is found from the figures that the influences of AO and WPSH represented a decay with distance, which is consistent with the reasoning advanced above. Also, the significant areas in the WTC of AO-rainfall showed an in-phase relationship between AO and monthly rainfall in January and April, representing a positive impact of AO on rainfall in the dry season and before flood season. It implies that monthly rainfall from these seasons would follow the change of AO. However, due to the effect of AO was weaker in the flood season (e.g. September) than dry season, a chaotic phase was found in the WTC graph of AO-precipitation in flood season. It implied that the monthly rainfall in the flood season could not always enhance when AO increased. Wen et al. [61] mentioned a similar conclusion. Zhou et al. [62] mentioned that the western Pacific subtropical high (WPSH) was closely related to the Asian climate and this effect would be stronger with the westward extension of WPSH.

Unlike this, WPSH had an irregular relationship with monthly rainfall as a whole, and only showed in-phase (positive influence) in September and non-significant anti-phase (negative effect) in January, Jul and Aug, demonstrating that rainfall in September would increase with the rising WPSH index and that would reduce in January, July, and August.

### Attribution of spatiotemporal variation of precipitation concentration indices

**Fig 10:**
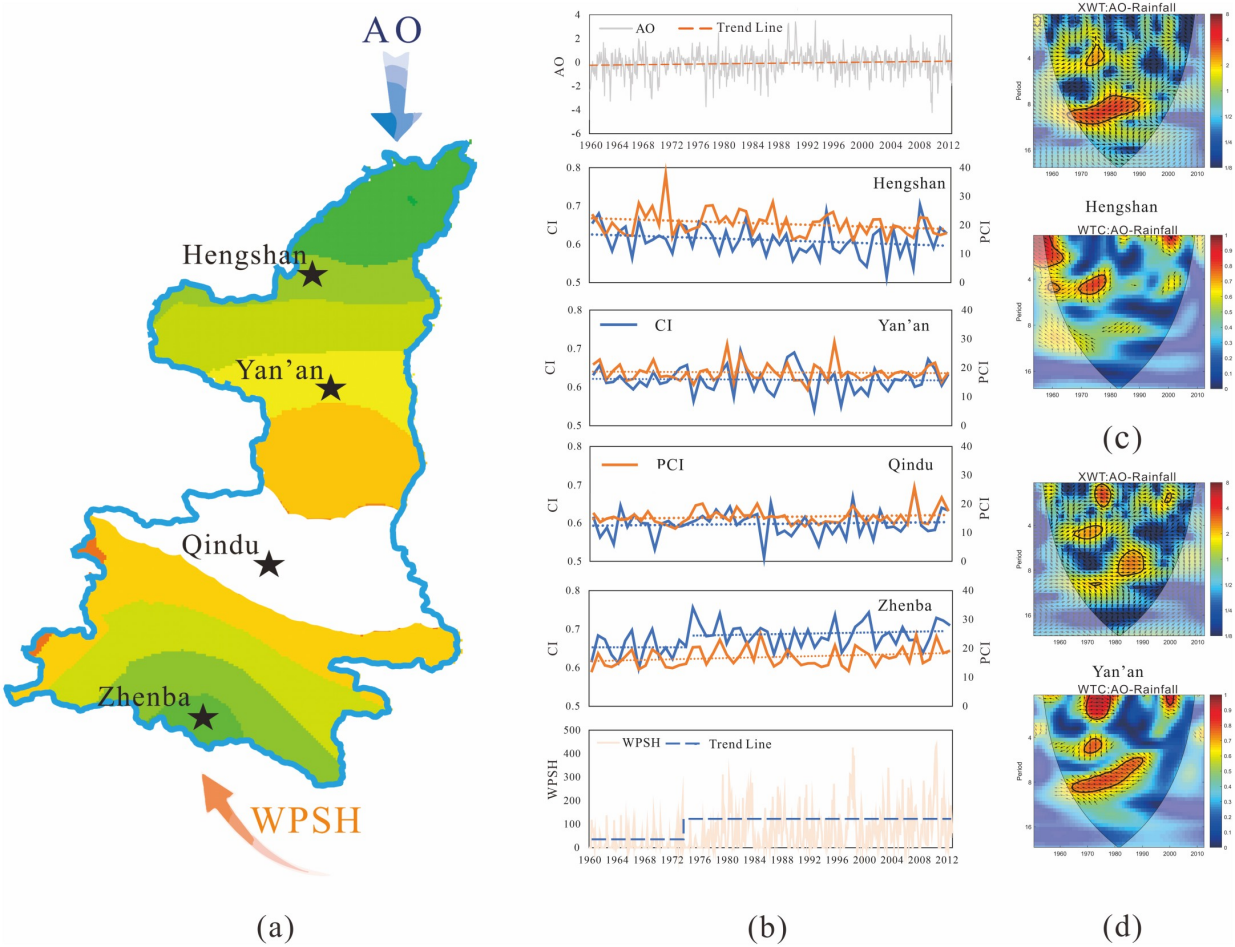
Movement (a) and trend changes (b) of atmospheric circulation indices, and changes in precipitation concentration indexes at meteorological stations (b), and AO-Rainfall XWT and WTC at Hengshan (c) and Yan’an (d) stations.

The impact of AO and WPSH on monthly precipitation has been fully discussed, and various effects have been identified in the section above. Considering the results of analysis of spatiotemporal variation of CI and PCI, a visualization of the influence of AO and WPSH on precipitation concentration is shown in Fig 10.

It can be observed from the figures that the AO and WPSH (Fig 10a) represent the increasing trend over time, WPSH of which enhanced greatly in recent years [63, 64], showing a noticeable abrupt change during 1972–1973 (Fig 10b). Considering the spatial position of four stations (Hengshan, Yan’an, Qindu and Zhenba) in Fig 10a and temporal variations of precipitation concentration indices mentioned above, the inference can be made that the increasing AO results in the precipitation rising in dry season and before flood season, and further brings the reduction of PCI in the affected area. Meanwhile, it is found from the XWT and WTC graph (Fig 10d) that the change of AO triggers the reverse change in precipitation in August when heavy precipitation often falls in a few rainiest days, and drives the CI decreasing in the affected area. To explore whether AO is a potential attribution of precipitation decreasing (including CI and PCI) in the study area, investigating the spatial differences of CI and PCI variations at Hengshan, Yan’an, and Qindu stations. It is seen that the influence of AO on precipitation concentration showed a decreasing characteristic with distance gradients, representing a layered or radial distribution from north to south, in which the obvious impacts on CI and PCI were basically invisible at Qindu station.

Additionally, Fig 10b shows that the varying WPSH has a significant influence on the variations in CI and PCI in the study area. Therein, the augment of WPSH drives rainfall in flood season with an in-phase and anti-phase change in the dry season, which causes the PCI index to increase in the dominant affected zone. However, the visible dominant impact on PCI was limited, only extending to Qindu station from the southernmost point of the study area. Focusing on the impact of CI variation, it can be found that WPSH plays a more important role in the variation of CI, representing similar changes with WPSH, such as an increasing trend and a jump in 1972–1973 at Zhenba station. But the visible influence was extremely limited spatially and undiscovered at Qindu station. It is considered that the dominant effect of WPSH on CI and PCI concentrated in southern Shaanxi.

In summary, the joint influence of AO and WPSH implied the attribution of changes in the temporal and spatial patterns of PCI and CI in the transitional zone between the Qinling Mountains and Loess Plateau. AO impacts the precipitation concentration and manifests a decreasing influence intensity with distance gradients from north to south, representing a layered or radial distribution. With AO increasing during 1960–2012, the PCI and CI in northern Shaanxi show a slowing reduction in the time domain, and the impacted change of two indices is also decreasing spatially with distance gradients in the transitional zone between Loess Plateau and Guanzhong Plain. Similarly, the influences of WPSH on precipitation concentration indices characterized the south-north layered variations of PCI and CI in southern Shaanxi, representing the concentration indices were increasing over time due to enhancing of the WPSH index and the augment delayed with distance gradients in the transitional zone between Qinling Mountains and Guanzhong Plain. Also, it should be noted that WPSH has a significant influence on the CI variation, demonstrating an in-phase and same-location jump change between them. In addition, it can be forecasted from the trend of AO and WPSH that the intensities of two indices could still increase in the future and precipitation concentration could be more significant in the area. Thus, flood prevention must be paid attention in the south part of the transitional zone between Qinling Mountains and Loess Plateau due to the storm rainfall events prone to flooding there, and water resources management in the north part should be carried out more effectively in the future due to more even monthly precipitation.

### Regional implications of precipitation concentration index

PCI represents the heterogeneity of monthly rainfall data, impacting regional or catchment water resources management (e.g. reservoir operation, water resources allocation, and water supply) and CI illustrates the weight of the days recording the largest daily rainfall event, implying the frequency and magnitude of flood and launching a challenge to flood control. It is considered that the investigation of regional implication of precipitation concentration indices is very significant to regional water resources safety and flood risk management. In this study, regional implication of CI has been mainly discussed, aiming to provide a reference for flood prevention in flood-prone areas of the transitional zone between the Qinling Mountains and Loess Plateau in recent years.

**Fig 11:**
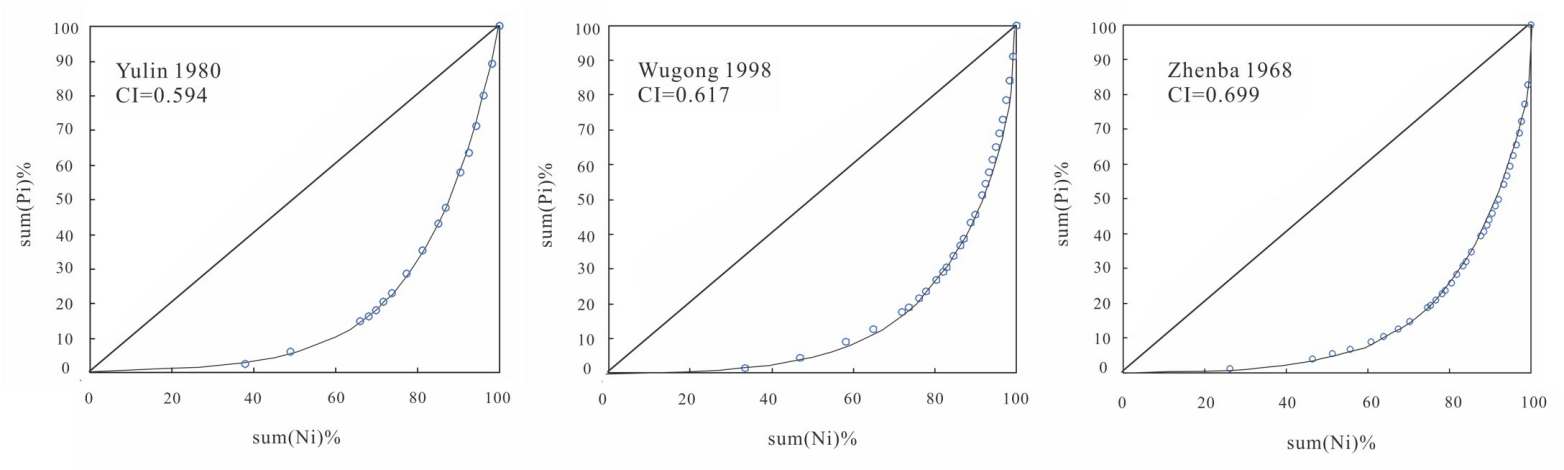
Lorenz curves of daily precipitation in certain year at three stations.

In the above-mentioned calculation of CI, Lorenz curves of daily precipitation in each year from 29 meteorological stations were drawn, and some of them are shown in Fig 11. They were not only the material for computing the CI indicator, but also represented the relationship between accumulated percentages of rainy days (∑*N*_*i*_ (%)) and the associative accumulated percentages of precipitation (∑*P*_*i*_ (%)). Martin-vide [39] proposed the CI value of 0.66 by analyzing Lorenz curves, aiming to mark some areas with CI over 0.66 in the central and western parts of Spain which had approximately more than 75% of the precipitation being contributed by 25% of the rainiest days. Although many researchers developed the application of CI in China [65, 66] and other countries/areas [67, 68], the local implication of CI was hardly discussed. To provide more local information for building a common standard of CI classification or degree criteria, the CI value implying 75%, 80%, and 85% of precipitation being contributed by 25% of the rainiest days was explored in this study.

**Fig 12:**
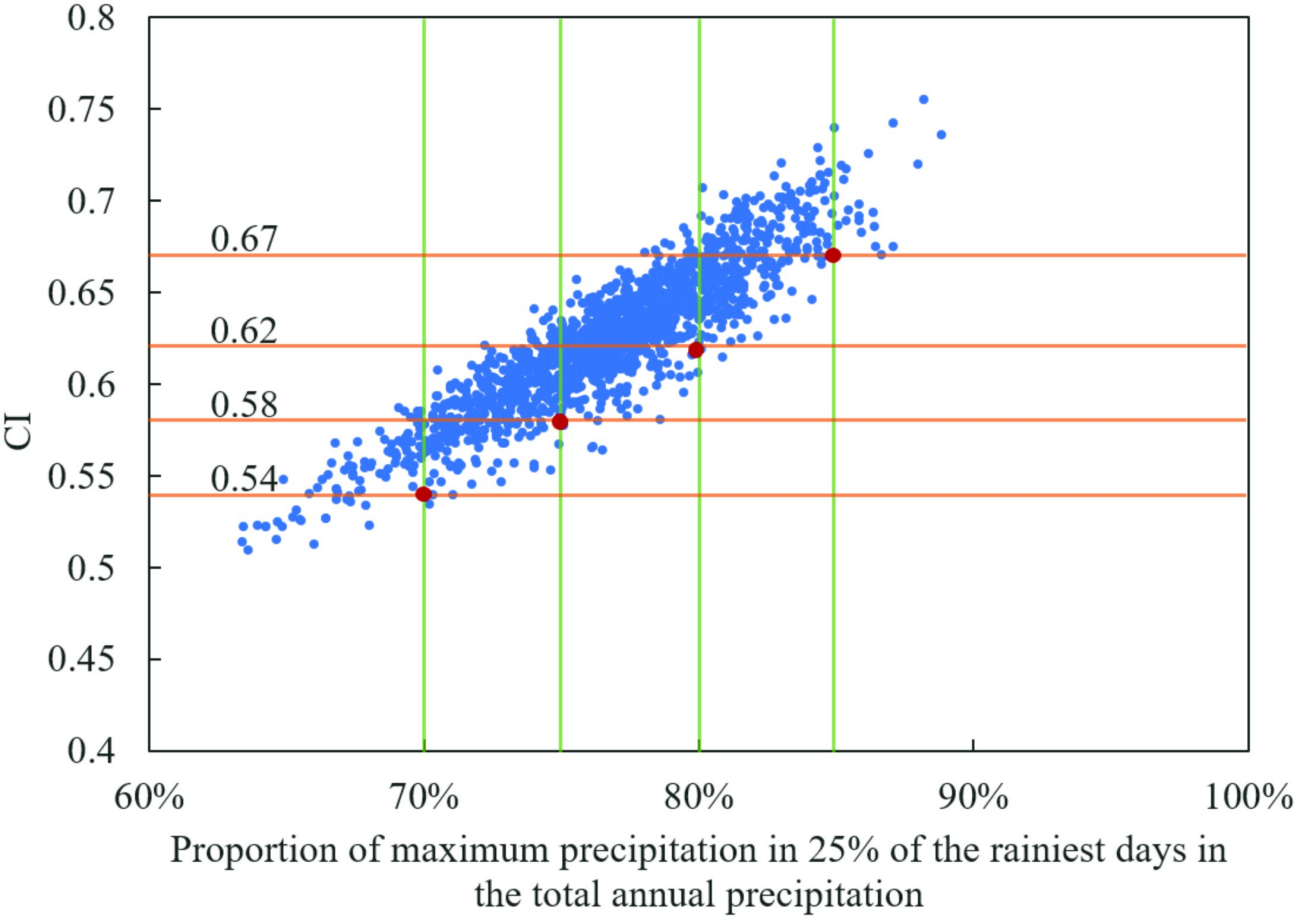
Scatter diagram between CI and the proportion of maximum precipitation in 25% of the rainiest days in the total annual precipitation.

To that end, a statistical analysis of over 2000 Lorenz curves of daily precipitation from all meteorological stations was performed, and the scatter diagram (Fig 12) was plotted between CI and the proportion of maximum precipitation in 25% of the rainiest days in the total annual precipitation. Through the diagram, CI can be determined, implying that 75% of the total precipitation amount occurred in 25% of the rainiest days. Analysis in Shaanxi Province indicated that when the CI value of an area was over 0.58, approximately 75% of the precipitation was contributed by 25% of the rainiest days in this region, different from the corresponding CI value in the central and western parts of Spain. When CI was over 0.62, approximately 80% of the precipitation occurred in 25% of the rainiest days, and CI was more than 0.67, implying that approximately 85% of precipitation fell in 25% of the rainiest days. Counting the areas with CI over 0.62 and 0.67 in Shaanxi Province from Fig 7a, it can be seen that 80% of the total precipitation amount basically occurred in 25% of the rainiest days in over 90% of the study area during 1957–1966. In the period of 1977–1986, the area reduced to about 50% of the study area, and there was a small area around Zhenba station where 85% of precipitation fell in 25% of the rainiest days. In 1997–2012, the area with 80% of the precipitation that fell in 25% of the rainiest days dropped down to about 30%, and the small area around Zhenba still existed. All of these indicated that the daily precipitation concentration overall decreased in Shaanxi Province, implying a positive trend of flash flood and debris flow control in the transitional zone between the Qinling Mountains and Loess Plateau, China. But a small area around Zhenba must be paid attention due to the storm rainfall events prone to flooding there.

## Conclusion

The study focused on precipitation concentration changes in Shaanxi Province, China, located in the transitional zone between Qinling Mountains and Loess Plateau, and investigated the changes in trend and the spatial distribution of monthly-scale precipitation concentration index (PCI) and daily-scale concentration index (CI), and analyzed the migration characteristics of the distribution over time. In addition, it discussed the implications of CI value in the Study area by statistical analysis, and explored the attribution of spatiotemporal changes of PCI and CI by detecting their relationships with atmospheric circulation indices, such as Arctic Oscillation (AO) and Western Pacific Subtropical High (WPSH). The main conclusions of this study can be summarized as follows:

1. Changes in annual precipitation. The annual precipitation in the study area demonstrated a layered distribution in the whole region, from 200–400 mm into the Mu US Desert in Northwest Shaanxi to more than 800 mm in the south area of Qinling Dabashan Mountains (QDM). Therein, the southern QDM was the area with the strongest variation in precipitation, representing an average growth of about 3–4 mm/km along the spatial gradient of precipitation. Mann-Kendall test results indicated that the decreasing precipitation covered the whole study area except for east-central QDM. However, it is noted that the reduction in the small area should be paid attention because it could make the Hanjiang to Wei River Water Diversion Project expose to risk, and would further cause the financial loss of the intake area when precipitation in primary water resources area and intake area would decrease significantly.
2. Changes in precipitation concentration index. The CI values, with slight variations, ranging from 0.6 to 0.66, exhibited a decreasing trend from south to north in Shaanxi Province. The CI at most stations showed a decreasing trend in the past 60 years, helpful to flash flood and debris flow control. But it should be noted that large floods in the central QDM could be more frequent than ever. The PCI values mainly varied from 14 to 23, illustrating a rising trend from the south region to the north region, contrary to the spatial distribution of CI. The PCI in the study area didn’t represent a significant change in trend on the whole. However, the decreasing change of PCI in the western QDM triggered an issue that when regional annual precipitation and PCI would both decrease greatly in the future, the water in the wet period can be diverted outside of the area, but could be less than what is expected in the project design. Thus, it is suggested that the water available in the wet period for the above-mentioned diversion project should be recalculated by considering PCI change and the risk be reevaluated. Also, the layered distribution was found in the mapping of PCI and CI, that of CI occurred in QDM, and that of PCI mainly occurred in the Loess Plateau in North Shaanxi (LPNS).
3. Attribution of precipitation concentration index change. The joint influence of AO and WPSH implied the attribution of changes in the temporal and spatial patterns of PCI and CI in the transitional zone between the Qinling Mountains and Loess Plateau. Spatially, AO mainly impacts the precipitation concentration in northern Shaanxi (LPNS), manifesting a layered or radial decreasing in influence on the intensity with the north-south distance gradient. The influence of WPSH on precipitation concentration characterizes the south-north layered variations of PCI and CI in southern Shaanxi (QDM). In central Shaanxi (Guanzhong Plain, GZP), the spatial distribution of precipitation concentration indices is chaotic and variable over time due to the joint influence of changing AO and WPSH. In the time domain, the increasing AO drives the slowing reduction in PCI and CI in LPNS, and the enhancing WPSH index causes the increasing of two indices and the jump in CI in recent years. In terms of the influence intensity, AO has a larger sphere of influence, but WPSH exhibits a greater strength of impact in the limited affected area. Considering the potential increase of AO and WPSH in the future, it is noted that flood prevention must be paid attention in the south part of the transitional zone due to the storm rainfall events being conducive to flooding there, and water resources management in the north part should be carried out more effectively due to more even seasonal precipitation.
4. The implication of precipitation concentration index. Statistical analysis indicated that the implication of CI values in the transitional zone between the Qinling Mountains and Loess Plateau was different from the original CI value from the central and western parts of Spain proposed by Martin-Vide. The CI value with over 0.58 indicated that approximately 75% of precipitation was contributed by 25% of the rainiest days in this region. The CI value with over 0.62 and 0.67 illustrated that approximately 80% and 85% of the precipitation occurred in 25% of the rainiest days, respectively. Using the precipitation concentration in the study area as a criterion, it was found that in over 71% of the study area, 80% of the total precipitation amount basically occurred in 25% of the rainiest days during 1963–1972, and the area reduced to about 34.28% in the period of 1983–1992, and the area dropped down to about 37.36% in 2003–2012. All of these indicated that daily precipitation concentration overall decreased in Shaanxi Province, implying a positive trend of flash flood and debris flow control in the transitional zone between the Qinling Mountains and Loess Plateau, China. But in a small area around Zhenba, the extremes must be paid attention due to the storm rainfall events being conducive to flooding there.

## Supporting information

S1 Data (XLSX)
